# A Quantum Ruler for Magnetic Deflectometry

**DOI:** 10.3390/e20070516

**Published:** 2018-07-09

**Authors:** Lukas Mairhofer, Sandra Eibenberger, Armin Shayeghi, Markus Arndt

**Affiliations:** 1 Faculty of Physics, University of Vienna, Boltzmanngasse 5, A-1090 Wien, Austria; 2 Fritz-Haber-Institut der Max-Planck-Gesellschaft, Faradayweg 4-6, D-14195 Berlin, Germany

**Keywords:** molecule interference, matter-waves, metrology, magnetic deflectometry, photochemistry

## Abstract

Matter-wave near-field interference can imprint a nano-scale fringe pattern onto a molecular beam, which allows observing its shifts in the presence of even very small external forces. Here we demonstrate quantum interference of the pre-vitamin 7-dehydrocholesterol and discuss the conceptual challenges of magnetic deflectometry in a near-field interferometer as a tool to explore photochemical processes within molecules whose center of mass is quantum delocalized.

## 1. Quantum Interference of Organic Molecules

In quantum mechanics we attribute both wave and particle properties to the basic entities of the theory, and following Louis de Broglie [1] we associate an oscillatory phenomenon of wavelength $\lambda_{dB}=h/mv$ to the center-of-mass motion of any particle of mass *m* and velocity *v*, even if it has a rich internal structure and exhibits internal excitations. This can be proven in a very intuitive way using matter-wave diffraction [2,3], in analogy to Young’s famous double slit experiment. In realizations with complex molecules the de Broglie wavelength is typically of the order of a few picometers while the molecular wave function can be delocalized by more than 10^5^ $\lambda_{dB}$, and hundreds or thousand times the size of the particle. At the same time a single, complex molecule can be composed of hundreds or even a thousand atoms, and each atom itself is composed of dozens of nuclei and electrons. This physical picture is complemented by acknowledging the presence of hundreds of vibrational modes and excited rotational states. At molecular temperatures around 500 K most of these modes are excited, leading to molecular rotation frequencies around $\Omega ≃2\pi \times 10^{9}$ rad/s and structural or conformational changes on the sub-nanosecond time scale. The molecules can thus be prepared in superpositions of position and momentum even though we can assign classical attributes such as internal temperatures, polarizabilities, dipole moments, magnetic susceptibilities and so forth to them. This philosophical aspect of macromolecular interferometry has very practical applications in metrology for the measurement of electronic, optical, and even magnetic molecular properties. Earlier work has shown that such parameters can be readily measured both in classical beam experiments [4,5] and in Talbot-Lau deflectometry [6,7,8]. Here, we propose that the de Broglie interference can also be a promising tool for photochemistry. The optically induced change of molecular geometry is often well-understood in solution, but little explored in the gas phase. We are interested in how such atomic rearrangements influence magnetic properties and study this for the example of 7-dehydrocholesterol (7-DHC) where isomerization causes a ring opening and a change of the particle’s magnetic susceptibility. We describe successful matter-wave interference with 7-DHC and a thought experiment that exploits the fact that modifications of the magnetic susceptibility will be seen as a relative shift of the de Broglie interference fringe pattern in an external magnetic field.

## 2. The Quantum Wave Nature of 7-Dehydrocholesterol

**Figure 1:**
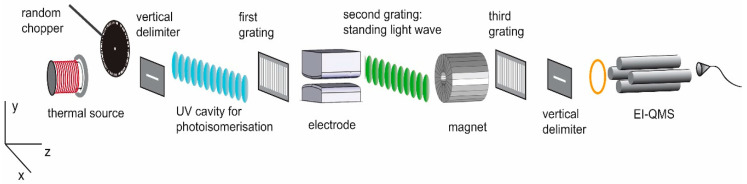
Sketch of Kapitza–Dirac–Talbot–Lau (KDTLI) interferometry, including the proposed extensions for magnetic deflectometry. The molecules evaporate to form a molecular beam in high vacuum. The molecular beam velocity is selected by its free-fall parabola using three horizontal slits. The molecular *v*-distribution can be recorded by chopping the beam in a pseudorandom sequence and measuring its arrival time at the quadrupole mass detector. The KDTLI comprises two nanofabricated absorptive masks, G1 and G3, and one optical phase grating G2. A tailored magnetic field (Halbach magnet) can exert a homogeneous force onto the molecules and deflect the molecular beam in proportion to the particles’ magnetic susceptibility. If the molecules exhibit a permanent magnetic dipole moment, the interference fringes will broaden, and contrast will be reduced.

Matter-wave physics with complex molecules is most conveniently realized using a near-field interferometer [9], for instance the Kapitza–Dirac–Talbot–Lau setup (KDTLI, see Figure 1), which is the basis here for our discussion [10]. This device is appealing for high-mass quantum experiments [10,11,12] since it is rugged, compact, and compatible with spatially incoherent particle sources. It has been used for demonstrating the quantum wave nature of organic molecules [13] even with masses beyond 10,000 amu [14] and as a tool for metrology [15,16]. Here we explore its potential for tracking optically induced changes in magnetic susceptibility.

The 7-DHC beam is prepared by sublimating molecules from a ceramic crucible at a maximal temperature of 460 K. Several delimiters shape a molecular beam of approximately 1 mm width and 200 µm height. They also select a well-defined velocity distribution $f\left(v_{z}\right)$ from an almost thermal initial beam. At the end of the setup, the molecules are ionized by electron impact and the ions are separated and counted by a quadrupole mass spectrometer. A mechanical chopper with a pseudo-random slit sequence imprints a time code onto the molecular beam and allows resolving its time-of-flight and velocity with a selectivity up to 1% [17].

**Figure 2:**
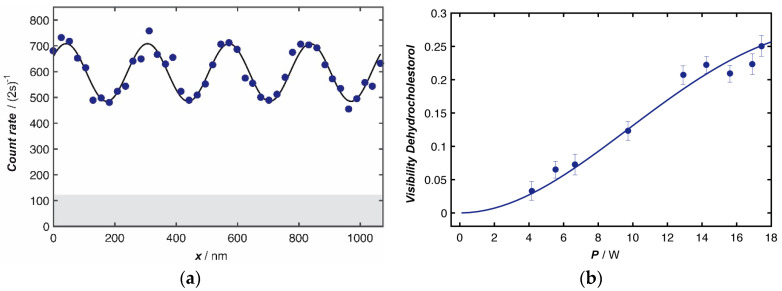
(**a**) Matter-wave interference of 7-dehydrocholestorol with a molecular beam velocity *v_mean_* = 212 ± 78 m/s (FWHM). The dots show the molecular count rate at the respective position of the third grating, and the continuous line is a sinusoidal fit to the data exhibiting a fringe contrast of 23.1 ± 1.5%. The grey shaded area indicates the dark counts of the detector; (**b**) The interference contrast varies with the laser power in the diffraction grating G2, following the line shape of the quantum model. We compared the achieved fringe contrast to the theoretical maximum by calibration measurements with the well characterized fullerene C_60_ and found a reduction of 10%, which we attribute to grating misalignment. This is still well compatible with fringe-assisted molecule metrology.

The interferometer consists of three gratings, all with a period of d=266 nm and positioned at equal distance L=0.105 m to one another. The first grating G1 is a periodic slit array in a 160 nm thick membrane of SiN_x_. Each slit is nominally 110 nm wide and the confinement of the molecular wavefunction in any slit suffices to expand its coherence function by several orders of magnitude further downstream. The center-of-mass wave function of wavelength $\lambda_{dB}$ diffracted at each slit of width s thus obtains a spatial coherence $W_{c}≃2\lambda_{dB}L/s$, which grows with distance to the source, such that the center-of-mass coherence function spreads over the extension of at least two slits when arriving at the second grating. The standing light wave G2 is obtained by retro-reflecting a 532 nm laser beam at a plane mirror. In the antinodes of the grating the light shifts the phase of the transmitted matter-wave mainly by the optical dipole potential, but the full quantum model includes absorption of photons as well [17]. At the center of the Gaussian laser beam, this phase depends on the power *P* and the vertical beam waist $w_{x}$ of the laser, as well as on the molecular optical polarizability α (532 nm), and the forward velocity of the molecule $v_{z}$. The coherent evolution of the molecules in phase-space leads to the formation of a molecular density pattern of period *d* = 266 nm, which can be sampled by the mechanical mask G3. This pattern forms periodically along the beam line and consecutive patterns are separated by the Talbot length $L_{T}=d^{2}/\lambda_{dB}$ [18]. Tracing the number of transmitted molecules as a function of the position of G3, one finds a nearly sinusoidal fringe pattern, as shown in Figure 2a with a visibility *V* = (*S_max_* − *S_min_*)/(*S_max_* + *S_min_*), where *S_max_* and *S_min_* are the maximal and minimal count rates.

In our experiments, 7-DHC had a mean de Broglie wavelength of $\lambda_{dB}≃4.9$ pm and showed a maximal fringe contrast of about *V* = 23%. Earlier experiments have shown that understanding such molecular density patterns requires quantum mechanics [3,10,11,12,19]. We confirm this here, by tracing the interference contrast as a function of the diffracting laser power (Figure 2b). While a fringe pattern could be mistaken as a classical Moiré shadow, the detailed dependence of the fringe visibility *V*(*P*) on the diffracting laser power can only be reproduced by quantum theory [18]. The quantum model assumes that the molecular wave function is delocalized over at least two nodes of the standing light wave, that is 200 times the molecular diameter, which has triggered philosophical discussions on the interpretation of quantum mechanics and the reality of the “position” of objects that we would see with 1 nm diameter in surface probe microscopy [20]. However, independent of this important question at the heart of physics, the predicted nanoscale molecular density pattern that arises as a consequence of quantum interference is an experimental fact, as shown in Figure 2a. And it is this nanoruler that we can use to extract even information about intra-molecular properties. Moiré deflectometers have been successfully used in the past to measure small forces on atoms [21] and they are interesting for advanced anti-matter experiments [22]. However, when aiming at higher force sensitivity and using smaller fringe periods such devices automatically become matter-wave interferometers which require quantum physics for a correct description.

## 3. Photo-Switching 

Photoactive molecules are interesting candidates for optically addressable memories, switches in organic electronics, and molecular motors [23]. Diarylethenes [24], fulgides [25], and spiropyrans [26] are common representatives. In solution, they are known to undergo photoisomerization associated with a ring opening or closure. Such photoisomerization is also known for resveratrol [27] and 7-dehydrocholestorol [28]. While most studies have been performed in solution, molecular beam experiments can shed light on the molecular excited state dynamics in a solvent-free environment. Photoisomerization in the gas phase has been demonstrated for spiropyran using electron-diffraction [29]. Here, we want to lay out a new perspective. 

**Figure 3:**
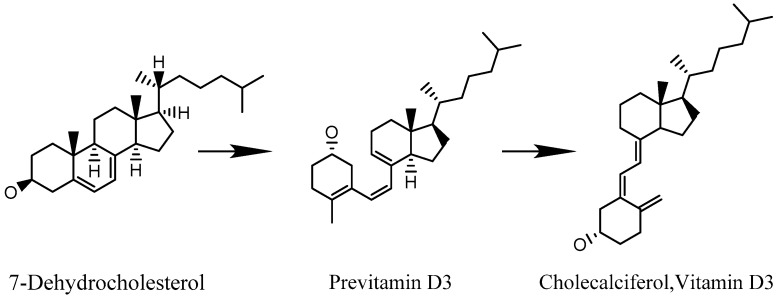
The photoisomerization (1) from 7-dehydrocholesterol (7-DHC, molecular weight MW = 384 amu) to previtamin D3 is well understood in solution, but little studied in the gas phase. This is also true for the spontaneous isomerization (2) from previtamin D3 to vitamin D3 (cholecalciferol).

In Figure 3 we show 7-dehydrocholesterol (7-DHC) as a prototypical molecule of biological relevance. It plays a vital role in the human metabolism and transforms into vitamin D3 via one photo-induced and one thermal isomerization process. The barrier for the required ring-opening is sufficiently high for the molecule to persist in closed-ring form, even when heated to 500 K.

When 7-DHC absorbs light in the wavelength range of 260–310 nm it can undergo photoisomerization, as shown in Figure 3 [30]. We assume the absorption cross section in solution $\sigma_{\mathrm{abs}}≃2\times 10^{-17}$ cm^2^ to be also a good approximation for molecules in the gas phase at T = 450 K. Recent experiments with photo-cleavable peptides showed that ultraviolet (UV) absorption cross sections of molecules in this complexity range can be comparable in the gas phase and in solution [31]. When a v=100 m/s fast 7-DHC molecule traverses a gaussian laser beam of power *P* and waist $w_{0}=0.3$ mm it will absorb on average $n=\frac{2P}{\pi w_{0}}\frac{\lambda }{hc}\frac{\sigma_{abs}}{v}$ photons. The average n=1 is reached for λ= 266 nm and P= 40 W. Single pass frequency doubling of a green solid state lasers can reliably generate ultraviolet light of *P* = 1 W and a power enhancement of 50–80 is conceivable in low finesse UV cavities, even in high vacuum where UV optics often suffer from outgassing [32]. Also, commercial high-power nanosecond lasers can produce up to 30 W average power at 266 nm and even 200 W at 355 nm, with repetition rates of 100 kHz. This is sufficient to ensure that all molecules interact with the laser beam. Positioned before the first grating, one or even two photoisomerization processes can be completed before the molecules enter the interferometer region. The following considerations focus on the feasibility of detecting such state changes via an interferometric monitoring of a change in molecular magnetism.

## 4. Magnetic Manifestations of Molecular Photoisomerization in the Gas Phase

Since the days of Stern and Gerlach, when magnetic deflection was used to demonstrate the discreteness of spin orientations [33], beam deflection experiments have become the basis for measuring atomic hyperfine structure [34], the realization of atomic clocks [35], or for studies of cluster magnetism [36,37]. The permanent magnetic moment of radicals has also been used to slow and cool beams of small molecules [38,39]. The magnetic manipulation of complex molecules is much harder to achieve, since their total orbital or spin angular momentum either vanishes or is too small in relation to the molecular mass. Here, we explore, whether the high force and position sensitivity of matter-wave fringes can provide additional information about the magnetic properties of molecules, which can also be a signature for photoisomerization processes.

To understand the different contributions to molecular magnetism, we invoke second order perturbation theory to distinguish the possible responses of a molecule to an external *B*-field [40,41]. This quantifies the energy shift of a molecule with vanishing total spin as

(1) $$\Delta E_{n}=\mu_{B}B\langle n\left|\Lambda \right|n\rangle +\frac{e^{2}}{8m_{e}}B^{2}\langle n\left|\underset{k}{\sum }\left(x_{k}^{2}+y_{k}^{2}\right)\right|n\rangle +\mu_{B}^{2}B^{2}\underset{n^{\prime }\neq n}{\sum }\frac{{\left|\langle n\left|\Lambda \right|n\prime \rangle \right|}^{2}}{E_{n}-E_{n^{\prime }}}.$$

Here *n* designates the electronic quantum number, *Λ* the quantum number of the projected angular orbital momentum, $\mu_{B}$ Bohr’s magneton, and *B* the modulus of the magnetic flux density. The mass and coordinates of the electrons are $m_{e}$, $x_{k}$ and $y_{k}$. The magnetic susceptibility $\chi_{mag}$ is the second derivative of the energy shift with respect to the magnetic field strength H, with $\left(H+M\right)\mu_{0}=B$, and $\mu_{0}=4\pi \times 10^{-7}\;N/A^{2}$ the vacuum permeability:

(2) $$\chi_{mag}=\frac{1}{\mu_{0}V}\frac{\partial^{2}\Delta E_{n}}{\partial H^{2}}.$$

The first term in Equation (1) represents the Langevin paramagnetic response for a particle with finite total angular momentum ***J****.* The magnetic moment $\mu_{J}$ interacts with the flux density ***B*** and experiences an orientation-dependent force $F=-\;\nabla \;\left(\mu_{J}\;B\right)$, which will pull an aligned magnetic dipole towards the field maximum and push the anti-aligned particle away. A thermal beam of molecules with random orientations of their figure and rotation axes will therefore be broadened, when exposed to a *B*-field gradient. In matter-wave interferometry, this broadening will reduce the interference fringe contrast. This resembles the observations for electric dipole moments in electric fields [8,42,43]. In the gas phase first order paramagnetism will always dominate over all other magnetic effects, unless the magnetic dipole moment vanishes. In the following we focus on those molecules, with ***J*** = 0 in the ground state.

The second term of Equation (1) represents the diamagnetic contribution. A diamagnetic molecule of susceptibility $\chi_{\mathrm{dia}}$ responds to an external B-field like a particle of polarizability *α* in an electric field. However, while an electric field induces and aligns a dipole moment such as to attract it to higher fields, according to $F_{el}=\alpha \left(E\nabla \right)E$, the induced magnetic moment will be expelled from regions of higher magnetic field strength with a force described by

(3) $$F_{dia}=-\beta \left(B\nabla \right)\;B$$

with $=\chi_{mol}^{dia}\;\mu_{0}^{-1}N_{A}^{-1}$, $\chi_{mol}^{dia}$ the molar diamagnetic susceptibility and $N_{A}$ the Avogadro number. The experiment will be sensitive to the orientational average of the magnetic polarizability, since the molecules will arrive with an isotropic distribution of initial orientations and rotation axes, and their rotation rate is fast compared to the transit time through the magnet.

The third term of Equation (1) is the second order contribution to paramagnetism, the van Vleck paramagnetism. The van Vleck force is often comparable in magnitude to the diamagnetic component but pointing in the opposite direction. 

Finally, in molecules we must also account for nuclear spins of different isotopes: Natural hydrocarbons contain ^13^C with an abundance of 1.1%. In natural fullerene for instance, 48% of all C_60_ molecules hold at least one nuclear spin and 10% even exactly two. In 7-DHC, still 26% of all molecules hold at least one nuclear spin. Since ^13^C has a nuclear spin of ½ and a nuclear magnetic moment of $\mu_{C13\;}=$ +0.7$\mu_{N}$ the nuclear response will be about two thousand times weaker than that of a single unpaired electron, but nuclear paramagnetism can actually be comparable to electron diamagnetism or van Vleck paramagnetism and must not be ignored for ***J*** = 0.

We set the scene by estimating the *B*-field configuration that is required to shift the interference pattern by 1/10 of the full interference fringe; i.e., by ≈26 nm. A constant force is achieved in a field of constant $(B\nabla )B_{x}$. The fringe deflection Δx depends on the molecular mass and velocity, the length $L_{1}$ of the magnet and the distance $L_{2}$ of its closest edge to G2, as well as on the total interferometer length L through the geometry factor *K =* $\left(L_{1}^{2}/2-L_{1}L_{2}+L_{1}L\right)$:

(4) $$\Delta x=K\frac{\beta }{mv^{2}}(B\nabla )B_{x}$$

We estimate the effect for isotopically pure ^12^C_60_ fullerenes whose magnetic response represents a lower limit to most of the interesting aromatic molecules. The molar magnetic susceptibility of C_60_ has been measured to be $\chi_{C60}=-1.08\times 10^{-9\;}m^{3}\cdot {\mathrm{mol}}^{-1}$ [44]. This translates into a molecular magnetic polarizability of $\beta_{C60}=1.4\times 10^{-27}{\;\mathrm{Am}}^{4}\cdot V^{-1}\cdot s^{-1}$. For *L* = 0.2 m, $L_{1}=\;0.04$ m, $L_{2}=$ 0.04 m, v=100 m/s, *m* = 720 amu, and *K* = 0.003, the interference fringe can be shifted by about 25 nm for $(B\nabla )B_{x}$ = 70 T^2^·m^−1^. If a field of that order of magnitude can be prepared, the fringe shift can still be resolved, the interferometer can still be sensitive to $\chi_{C60}$. The case of fullerene C_60_ gives a conservative limit, since the deflection depends on the magnetic polarizability-to-mass ratio β/m. For example, the fully aromatic molecule benzene C_6_H_6_ exhibits five times greater β/m.

While a full quantum chemical assessment of the magnetic properties of 7-DHC exceeds the scope of this work, we expect the ring opening to induce magnetic susceptibility changes to be at least on the order of the effect estimated here. Since the fringe shift grows linearly with the interferometer length and quadratically with the length of the magnet, future long-baseline interferometers will be ten times more sensitive, at least, and certainly allow measuring even such tiny magnetic susceptibilities.

## 5. Design of the Required Magnetic Structures 

**Figure 4:**
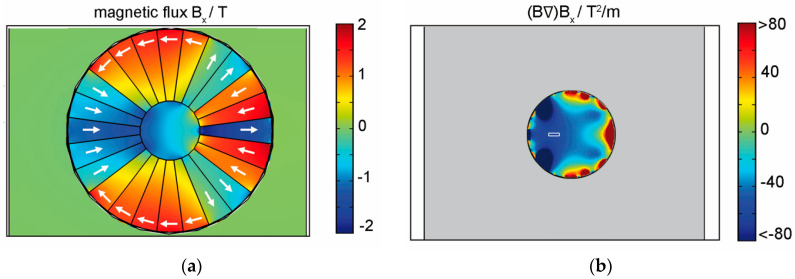
Finite element simulation of the modified Halbach cylinder. (**a**) Magnetic flux: the arrows show the direction of magnetization of the individual segments; (**b**) magnetic force field: the deflection of a molecular beam is proportional to $(B\nabla )B_{x}$. The diameter of the magnet is 55 mm with an inner bore of 16 mm. The white rectangle indicates the location of the molecular beam, where the force is constant within 2%.

Such a high (B∇)B field can be realized using a modified Halbach cylinder, as shown in Figure 4, which we have simulated using the finite element package COMSOL 4.0 multiphysics simulation package (COMSOL AB, Stockholm, Sweden). The arrangement of permanent magnets from neodymium-iron-boron alloy, with a remanent magnetization of 1.3 T and a coercitive field strength of 100 kA/m, can guide the field lines inside the cylinder and generate the required field. Figure 4b shows that one can realize a region with $(B\nabla )B_{x}$ = 70 T^2^/m that is homogeneous within 2% of its peak value across an area of 1000 × 200 µm^2^; i.e., across the full molecular beam profile inside a KDTL interferometer.

## 6. Discussion

Our experimental data demonstrate that complex, thermal biomolecules can show quantum interference and be delocalized by a few hundred times their own size. We have also seen that the free-flying molecular nanostructure is a sensitive ruler to measure interference fringe displacements, which can quantify internal molecular properties in the presence of external perturbations. Here we have focused on the role of magnetic fields and showed that even very small magnetic contributions can become accessible in matter-wave assisted deflectometry.

**Figure 5:**
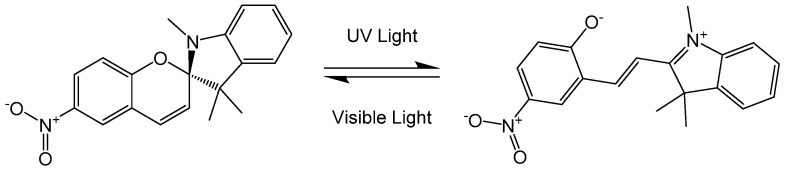
Spiropyran can isomerize to merocyanine upon absorption of a UV photon. This opens one ring which we expect to significantly change the magnetic susceptibility. In contrast to the case of 7-DHC, the process changes the electric dipole moment here by a large factor, from 7 Debye for spiropyran [46] to between 20–50 Debye for merocyanine [26]. Such huge changes will be easily detectable in interferometric electric deflectometry [8].

This can open an entire new range of experiments with photo-isomerization groups in spiropyrans, fulgids, and diarylethenes. Spiropyran, for instance, isomerizes to blue merocyanin upon absorption of a UV-photon around 365 nm and the reaction can even be reversed by irradiation with visible light [45]. See Figure 5.

Merocyanine is zwitterionic with a large electric dipole moment [26] and the isomerization should also be readily detected in interference-assisted electric deflectometry. Thus, a combination of electric and magnetic deflectometry will give insights into the molecular dynamics in the gas phase. Since spiropyrans can thermally isomerize to merocyanine above room temperature [45], optical switching experiments will be best performed with internally cold molecules [47]. The scheme can be generalized to a wide class of molecular systems.
